# Mechanisms of Drug Resistance in the Pathogenesis of Epilepsy: Role of Neuroinflammation. A Literature Review

**DOI:** 10.3390/brainsci11050663

**Published:** 2021-05-19

**Authors:** Elena D. Bazhanova, Alexander A. Kozlov, Anastasia V. Litovchenko

**Affiliations:** 1 Sechenov Institute of Evolutionary Physiology and Biochemistry, Russian Academy of Sciences, 194223 St. Petersburg, Russia; anastasiya_litovchenkos@list.ru; 2 The Federal State-Financed Institution Golikov Research Clinical Center of Toxicology under the Federal Medical Biological Agency, 192019 St. Petersburg, Russia; Alexandrk0zlov89@yandex.ru

**Keywords:** drug-resistant epilepsy, epileptogenesis, neuroinflammation

## Abstract

Epilepsy is a chronic neurological disorder characterized by recurring spontaneous seizures. Drug resistance appears in 30% of patients and it can lead to premature death, brain damage or a reduced quality of life. The purpose of the study was to analyze the drug resistance mechanisms, especially neuroinflammation, in the epileptogenesis. The information bases of biomedical literature Scopus, PubMed, Google Scholar and SciVerse were used. To obtain full-text documents, electronic resources of PubMed Central and Research Gate were used. The article examines the recent research of the mechanisms of drug resistance in epilepsy and discusses the hypotheses of drug resistance development (genetic, epigenetic, target hypothesis, etc.). Drug-resistant epilepsy is associated with neuroinflammatory, autoimmune and neurodegenerative processes. Neuroinflammation causes immune, pathophysiological, biochemical and psychological consequences. Focal or systemic unregulated inflammatory processes lead to the formation of aberrant neural connections and hyperexcitable neural networks. Inflammatory mediators affect the endothelium of cerebral vessels, destroy contacts between endothelial cells and induce abnormal angiogenesis (the formation of “leaky” vessels), thereby affecting the blood–brain barrier permeability. Thus, the analysis of pro-inflammatory and other components of epileptogenesis can contribute to the further development of the therapeutic treatment of drug-resistant epilepsy.

## 1. Introduction

Neuroinflammation is mediated by the synthesis of cytokines, chemokines, reactive oxygen species and secondary messengers. These mediators are produced by the central nervous system (CNS) resident glia (microglia and astrocytes), endothelial cells and peripheral immune cells. Neuroinflammatory reactions lead to immune, physiological, biochemical and psychological consequences. The purpose of the study was to analyze the drug resistance mechanisms, especially neuroinflammation, in epileptogenesis, as well as the currently available hypotheses of the drug resistance of epilepsy. Neuroinflammation aspects vary depending on the disease, trauma, exposure to toxic substances, infection or stress experienced [1]. Diseases with an autoimmune component such as optic neuromyelitis and multiple sclerosis [2], aging and obesity [3] can also be considered triggers of inflammation. Accumulating evidence suggests that neuroinflammation plays an important role in the etiology of mental disorders, a variety of neurological and somatic diseases, including epilepsy, Alzheimer’s disease, Parkinson’s disease, autism spectrum disorders, bipolar disorder, affective disorders, depression [4,5,6,7,8] and systemic lupus erythematosus [9,10]. Recent data suggests that neuroinflammation is one of the key processes in glaucoma and retinopathy [11,12,13,14], it is also important in the pathogenesis of heart failure [15]. Frequent or chronic inflammation can also have dangerous consequences, contributing to the development of malignant neoplasms [16] or to the metastasis of cells of existing tumors to the brain [17]. Recent immunological studies in psychiatry have led to the hypothesis of mild encephalitis (ME) in the pathogenesis of severe mental disorders. The ME hypothesis assumes the course of mild neuroinflammation, which is rather difficult to detect using available diagnostic methods [18]. The definition of autoantibodies targeting synaptic and neuronal cell surface proteins (NMDAR, AMPAR, GABAßR, etc.) in ME reflects its autoimmune nature, which has helped diagnose psychiatric autoimmune encephalitis. The presence of these autoantibodies was manifested mainly by psychiatric symptoms (psychosis), while neuroinflammation was proven in brain biopsies, and the so-called subtle epilepsy was also diagnosed using electroencephalography (EEG) [19]. Immunomodulatory therapy reduces the incidence of psychosis in autoimmune encephalitis [20]. Thus, neuroinflammation is an integral feature of many neurological disorders. Neuroinflammation does not mean a poor prognosis and some aspects of the neuroinflammatory response may be useful for restoring the CNS, for example, in the axon regeneration and their remyelination in the cases of multiple sclerosis, traumatic spinal cord injury, stroke and Alzheimer’s disease. Conclusions that neuroinflammation may be beneficial should not be surprising [21].

## 2. Materials and Methods

In preparing the review, the main and most famous information bases of biomedical literature, Scopus (766), PubMed (1419), Google Scholar (17 300) and SciVerse (192), were used. To obtain full-text documents, electronic resources of PubMed Central (PMC) and Research Gate were used. The text of the review cites 87 contemporary publications for 2014–2021, reflecting the latest discoveries, as well as 2 publications for 2008 and 2009. The search was carried out for the following words and phrases: drug-resistant epilepsy, refractory epilepsy, epileptogenesis, neuroinflammation. The topic of the selected articles was as close as possible to the topic of this article. Preference was given to articles in English with high citation, published in journals with a high impact factor in 2019–2021. The selection of sources included both individual independent studies and review articles with open access which examined the nature and manifestations of drug-resistant epilepsy in animals and humans, since the data for humans and experimental animals may differ, which offers evidence for the hypotheses considered. For selected articles, the number of published articles by the author in the given research area, the total number of citations of the author’s publications, the total number of publications cited by the author and the Hirsch index were estimated.

## 3. Epilepsy, the Characteristics of the Pathogenesis, the Relationship with Inflammatory Processes. Drug-Resistant Epilepsy

Epilepsy is a chronic neurological disease characterized by recurrent spontaneous seizures. Epilepsy affects approximately 1.0% of the world’s population [22]. The prevalence and frequency of epilepsy is slightly higher in men compared to women. The general prognosis of epilepsy is favorable for the majority of patients and 55–68% of them tend to achieve long-term remission [23], but in the case of generalized tonic-clonic seizures, nocturnal seizures and refractory or drug-resistant epilepsy, death is not uncommon [24]. The development of epilepsy can be associated with a wide variety of factors, including genetic predisposition, malformations, traumatic brain injuries that occur annually in 64–74 million people [25], chemical exposure, hypoxia or stroke. Many of these pathologies are associated with neuronal death. In 1971, M. Taylor discovered a disorder of the neocortex organization in patients operated on for drug-resistant epilepsy. Later, this disorder was called focal cortical Taylor dysplasia. Histologically, with Taylor’s dysplasia, there is an increase in the number of the cerebral cortex layers or their thickness, the presence of giant and dysmorphic neurons, “balloon cells” and heterotopy of neurons in the subcortical white matter [26]. Focal cortical dysplasia can be detected in almost half of patients with drug-resistant epilepsy [27]. Brain damage triggers a cascade of biological events characterized by neuroinflammatory processes—the release of cytokines, chemokines, lipid mediators and proteins in the neuronal microenvironment. In the brain, such mediators activate microglia and astrocytes, alter cerebrovascular function, affect the infiltration of peripheral inflammatory cells, promote cell proliferation or apoptosis and alter ion transport, neurotransmission and communication between neurons [28].

Currently, the role of complement in the pathogenesis of epilepsy has been shown. Complement activation can be caused by necrotic cells, cellular fragments or a misfolded protein such as the fibrillar form of amyloid β-peptide in Alzheimer’s disease [29]. Activated microglial and astrocyte cells synergize with other pro-inflammatory cascades, thus accelerating pathogenesis and neuronal dysfunction [30]. The complement includes about 30 plasma and cell membrane proteins that interact with each other, triggering a series of inflammatory responses involved in protecting against infection [31]. Dysregulation of the classical (C1inh, C4), alternative (FH, properdin, C3) and terminal (TC) pathways also contributes to the pathogenesis of epilepsy [31]. Overexpression of various complement proteins has been demonstrated in surgically removed tissue of patients with epilepsy, as well as in rodent models [32,33].

Cytokines are mainly produced by microglia and astrocytes in the CNS. They play a role in its development and normal functioning, as well as in numerous inflammatory reactions in neurodegenerative diseases [31,34,35]. Some regulation mechanisms of cytokine secretion and their receptors expression have been described in patients with epilepsy [36]. For example, increased concentrations of serum IL-1b, IL-1Ra, IL-2, IL-4, IL-6, IL-8, IFNy and IL-17 have been observed in patients with epilepsy [37,38], as well as increased levels of IL-6 and IL-17 in the cerebrospinal fluid [39]. Several researchers have shown that the concentration of IL-4, IL-8 and IL-17 can correlate with the frequency and severity of seizures, suggesting a key role for cytokines in the diagnosis and treatment of epilepsy [38,40]. Most chemokines and their receptors promote neuroinflammation by involving peripheral monocytes and promoting glial cell activation [25]. Table 1 provides examples of some mediators involved in neuroinflammation and epilepsy and their role in these processes.

Focal or systemic unregulated inflammatory processes lead to the formation of aberrant neural connections and hyperexcited neural networks [46]. There is a correlation of prevalence between autoimmune diseases and epilepsy; the risk of epilepsy is 3.8 times higher in people with one of the 12 autoimmune diseases and in children it is higher than in adults [31]. Despite the effectiveness of anti-seizure medications (ASMs) and the recent introduction of new ASMs, adequate treatment cannot be found in almost 30% of epilepsy cases. The drug resistance definition did not remain unchanged. In 2017, the International League Against Epilepsy (ILAE) proposed the definition of drug resistant epilepsy as the presence of seizures within 6 months, even with proper therapeutic regimens with one or more drugs [47]. The only thing that remains unchanged is that it is possible to detect drug resistance in clinical practice only after unsuccessful courses of treatment with several ASMs. At the primary diagnosis, it is very difficult to predict whether and how high the risk of developing such epilepsy is, excepting of certain diseases, such as West’s syndrome or Lennox–Gastaut syndrome. A poor prognosis is more likely to be expected in people with mental retardation and serious deviations from the norm on the electroencephalogram [48].

Neurosurgical resection of the epileptic focus [49] or neurostimulation [50,51] can help such patients. These methods reduce the seizure frequency by 10–80%. It is considered promising to switch to a ketogenic diet, for example, the modified Atkins diet [52], which has been used since 2003 to treat children and adults with refractory epilepsy at Johns Hopkins Hospital in Maryland, USA [53]. Other dietary regimens, such as calorie restriction and a gluten-free diet, can also have a positive effect [54]. In the absence of an effect, drugs based on cannabidiol or its synthetic analogues can be prescribed as palliative care, reducing the number of epileptic seizures in about 80% of patients but the frequency of side effects is high—from 42 to 71.4% [55,56]. Drug resistance is not an absolute category. Six trials involving 2411 people aged 16 to 80 years showed that treatment with the third-generation ASMs Brivaracetam, a high-affinity ligand for the synaptic vesicle protein 2A, showed a decrease in seizure frequency by 50% and more, compared with placebo groups [57]. Most seizures, even in the case of drug-resistant epilepsy, stop spontaneously. In particularly severe cases of so-called “super-refractory epilepsy”, when the seizure lasts 24 h or more and there is a risk of irreversible damage to neurons and their death, almost all experts use “aggressive” therapy with continuous intravenous infusions of midazolam, pentobarbital or propofol in the intensive care hospital department. In this case, the seizure can be stopped almost always but the overall mortality rate can reach 48%. About one fifth of patients with refractory epilepsy are at risk of this [58].

## 4. Hypotheses of the Causes of Drug Resistance of Epilepsy

Early diagnosis of refractory epilepsy allows immediate prescription non-drug treatment for such patients. The identification of such patients is currently difficult. Most of the data concerning epileptogenesis, the role of the inflammatory process and drug-resistant forms of epilepsy have been obtained in animal models. So, two models of epilepsy: West’s syndrome induced by intracerebral injections of proinflammatory compounds and infection with the Tayler mouse encephalomyelitis virus, have shown a large role of inflammatory processes in the epilepsy pathogenesis. A common feature of these models was the drug resistance profile [59]. There are several theories about the nature of drug resistance, none of which individually explains its neurobiological basis [50,60].

1. According to a pharmacokinetic hypothesis, overexpression of drug efflux vectors in peripheral organs lowers the levels of ASMs. This does not allow drugs in sufficient concentration to pass into the brain to the epileptic focus. This theory is based on clinical observations when the decrease in the concentration of ASMs could not be explained by the overexpression of P-glycoprotein (Pgp), multidrug resistance protein 1 (Pgp, MDR1; CD243) or other transporters on the BBB and in neurons [50]. The role of Pgp in the refractory epilepsy genesis is undeniable and experiments on rats have shown that suppression of the miR-146a gene can attenuate pathological changes and reduce drug resistance in refractory epilepsy [61]. Changes in the expression and functionality of multiple drug carriers in patients with refractory epilepsy do not have to be limited to the brain but can also occur in other tissues, such as the small intestine or kidney. In addition, in humans, the metabolism of ASMs is mainly mediated by liver cytochrome P450. Some of the cytochromes of this group have allelic types encoding isoforms which have different activity and, in turn, can affect the concentration of many drugs, including ASMs, in the blood serum [62]. The animal studies do not support the pharmacokinetic hypothesis [60].
2. The transport hypothesis is similar to the pharmacokinetic hypothesis. According to the transport hypothesis, overexpression of drug efflux carriers in drug-resistant epilepsy occurs directly in the BBB and not at the periphery, leading to a decrease in drug absorption by the brain and, as a consequence, to the resistance [50]. The role of carriers of the efflux of several drugs, such as Pgp, has been studied, as well as their effect on the regulation of drug penetration through the BBB into the brain [63]. It is known that overexpression of carriers of the efflux of several drugs was one of the reasons for the failed attempts to treat some cases of brain tumors or neuroinfections [64]. Pgp synthesis is regulated by the ABCB1 gene. Its expression is of clinical importance, since Pgp has a wide substrate specificity, it affects the binding to drugs, which differ significantly in their chemical structure [65]. It was shown that Pgp, together with other proteins from the group of multidrug resistance, is overexpressed in the endothelial cells of the brain capillaries and in the astrocytes of patients with drug-resistant epilepsy [66]. The endothelial barrier function of the BBB is temporarily and locally impaired during seizures [67], which, together with the overexpression of several drugs carriers in the astroglia covering the blood vessels, can have a double barrier effect for the pervasion of ASMs and reduce their extracellular concentration. Thus, epilepsy caused by the presence of this pathology becomes resistant to treatment due to insufficient concentration of drugs. Studies in animal models show that inflammatory proteins activate the expression of carriers of ATP-binding cassettes, which are responsible for drug resistance, but this remains unclear in human tissues. There are also potential links between inflammatory markers such as cyclooxygenase enzymes COX-1, COX-2 and the 18 kDa translocator protein (TSPO) expressed in microglia and efflux transporters [68].
3. The neural network hypothesis suggests that due to neuron degeneration and the synaptic network remodeling, the brain’s seizure control system is suppressed and drug access to targets is restricted [50]. Changes in the neural network are a fundamental mechanism of cognition, perception and consciousness. Network activity disturbances play a crucial role in the pathophysiology of brain diseases. The development of non-invasive neuroimaging techniques and machine learning technologies have made it possible to test this hypothesis. It has been shown that individual brain models of fifteen patients with drug-resistant epilepsy obtained on the basis of diffuse magnetic resonance imaging have prognostic power [69]. Cortical dysplasia is often associated with drug-resistant epilepsy [70]. It is proved that the pathology of neural networks underlies focal drug-resistant epilepsy and such methods as cathodic transcranial direct current stimulation, which change the connectivity of the epileptic focus, reduce the frequency of seizures in patients who were not helped by surgical treatment [71]. The concept of epileptic networks has also been confirmed in a rare form of focal epilepsy called sleep hypermotor epilepsy (SHE). The functional connectivity of the sensorimotor cortex and thalamus in thirteen SHE patients was higher than in thirteen healthy [72]. Experimental evidence shows that the hippocampus is associated with drug resistance in a rat model of drug-resistant epilepsy [73]. The main weakness of this hypothesis is that not all patients with cortical dysplasia and changes in the neural network exhibit refractoriness [50,74]. In addition, not all treatment-resistant patients respond to ASMs after temporal lobe resection, even with complete EEG-susceptible resection of the epileptogenic zone [75].
4. The intrinsic severity hypothesis suggests that common neurobiological factors affect both the severity of epilepsy and drug resistance. Clinical reports support the intrinsic severity hypothesis, showing that high pretreatment seizure rates are an important predictor of refractory epilepsy [50]. A transcriptome analysis of hippocampal tissues removed from patients with mesial temporal lobe epilepsy, the most common form of focal epilepsy observed in 40% of adult patients and resistant to ASMs in 30% of cases, was performed. This analysis showed aberrant expression of three important gene clusters, these genes are mainly associated with neuroinflammation and innate immunity, synaptic transmission and neural network modulation. These results support the hypothesis of intrinsic complexity of drug resistance. Randomized clinical trials of children and adults have shown that starting treatment after the first tonic-clonic seizure does not improve prognosis of epilepsy and the probability of resistance does not depend on the number and severity of seizures before treatment [50].
5. The genetic variants hypothesis states that polymorphisms are associated with pharmacodynamics, metabolic pathways, enzymes, ion channels and neurotransmitter receptors, block drug binding, metabolism and transport. Most commonly, there are: gene 1 of subfamily B of ATP-binding cassette (ABCB1 or MDR1) and subfamily C of ATP-binding cassette (ABCC2 or MRP2), subunits 1, 2 and 3 of potential-dependent sodium channels (SCN)—SCNα (SCN1, SCN2 and SCN3); metabolizers of endogenous and xenobiotic substances, cytochrome P450 families 2 and 3 (CYP2 and CYP3), genes for acetylcholine receptors, neural potassium channels, calcium channels and GABA receptors [76]. Recent data supports the important role of genetics in patients with untreatable seizures. Next-generation sequencing technologies have increased the diagnostic value of genetic analysis from 10% a few years ago to 30–40% today. The number of genes in existing commercial panels already reaches hundreds and whole-exome sequencing allows us to identify new single nucleotide polymorphisms, including “effective” genes, when corrective therapy can significantly reduce the number of seizures or stop them [77]. Polymorphisms of genes encoding channels, receptors, transporters, synaptic transmission, etc., were associated with various types of epilepsy, and some were associated with refractory epilepsy [78].
6. The epigenetic hypothesis has been developed in recent years. An epigenome is a set of molecules that regulate the gene expression in the genome. Unlike a more or less fixed genome, the epigenome is dynamic and its changes can explain the change in drug resistance patterns. The study of the epigenomic contribution to drug resistance in epilepsy is an extremely complex task, in which it is difficult to separate cause from effect and significance from epiphenomena [60,79]. Manipulations with specific microRNAs can affect convulsions and the course of disease in laboratory animals but there is very little data on humans, especially without genetic abnormalities [80]. A study of 75 people from northern China, 25 of whom had carbamazepine (CBZ)-resistant epilepsy, showed a significant difference in methylation levels in the promoter of the epoxide hydrolase 1 gene EPHX1 between them, CBZ-sensitive patients and controls. In CBZ-resistant epilepsy, a methylation increase was observed in the region of the promoter NC_000001. 11 (225, 806, 929, …, 225, 807, 108). There was a significant positive correlation between the seizure frequency, the course of the disease and the methylation of EPHX1 in the CBZ-resistant group [81]. Analysis of DNA methylation across the entire genome and gene expression in brain tissues in 10 patients with refractory epilepsy showed the presence of many differentially methylated genes on X chromosome and a significantly smaller number on Y chromosome. Sixty-two differentially expressed genes, such as MMP19, AZGP1, DES and LGR6, were first correlated with refractory epilepsy [78].
7. The target hypothesis postulates that changes in the properties of drug targets, such as changes in potential-dependent ion channels and neurotransmitter receptors (for example, the GABA receptor), lead to a decrease in drug sensitivity. According to this hypothesis, changes in voltage-gated ion channels in Dravet syndrome and neurotransmitter receptors, lead to a decrease in drug sensitivity and refractoriness. This hypothesis was based on the study of the effectiveness of carbamazepine and phenytoin, but it was not demonstrated what happens with other drugs that block sodium channels. Many but not all ASMs prevent seizures by blocking potential-dependent sodium channels of the brain [50,60,82]. In addition, patients with drug-resistant epilepsy usually do not respond to drugs of different classes with different mechanisms of action and in this case, the reason may lie in unknown non-specific mechanisms of resistance.
8. The hypothesis of neuroinflammation is the most attractive. In the experiment and in clinic, it was found that the permeability of the BBB increases in foci of chronic epilepsy. Artificially induced dysfunction of the BBB induces the appearance of epileptic foci in a previously healthy brain [83]. The artificially induced BBB dysfunction is associated with the induction of Pgp in the cerebral vessels and astrocytes, as described above, while these disorders were accompanied by a neuroinflammatory response in the same areas of the brain where the epileptic focus appeared. Neuroinflammation can be an inducer of BBB dysfunction and an increase in Pgp regulation in drug-resistant epilepsy. It has been suggested that inflammatory mediators can induce drug-resistant seizures in three ways. One of these is by a direct effect on the endothelium of cerebral vessels, including by direct destruction of tight contacts between endothelial cells, induction of abnormal angiogenesis, expressed in the formation of “leaky” vessels and oxidative stress [83]. A similar effect destroying the integrity of the BBB can be caused by the inflammatory activity of astrocytes and, conversely, changes in the BBB permeability can promote the expression of inflammatory molecules in astrocytes [84]. This vicious circle promotes seizure recurrence, cell loss and maladaptive plasticity of neural networks. Penetrating into the nervous tissue through the damaged BBB, serum albumin, which normally should not be present there, increases the drug binding effect, thereby reducing functionally significant levels of unbound drugs in the target brain regions [85]. Another mechanism of the effect of neuroinflammation on the development of epilepsy resistance is stimulation by proinflammatory mediators of Pgp in endothelial cells, which may confirm the transport hypothesis of drug resistance [86]. The third pathway involves post-translational modification in voltage-dependent ion channels by inflammatory mediators, which leads to a decrease in the sensitivity of these receptors to the drugs used [87].

These hypotheses are summarized in Table 2.

The fact that at least four types of drug resistance can be observed in clinical practice does not strengthen the position of each of the presented hypotheses:

1. de novo resistance, in which the patient never enters a useful period of absence of seizures from the onset of epilepsy.
2. delayed resistance, when the seizures initially stop but then return and become uncontrollable.
3. fluctuations in resistance with an increase and decrease, when epilepsy is alternately controlled and not controlled by the same drug or a combination of them. This course of epilepsy does not support the hypothesis of internal severity [88] and some others.
4. epilepsy which is initially resistant to drugs but eventually responds to treatment [60].

## 5. Conclusions

Despite the emergence of various new ASMs with different mechanisms of action, drug resistance remains one of the main problems in the treatment of epilepsy. In the case of an individual patient, resistance is mediated by several mechanisms, the contribution of which may vary.

Overcoming resistance will not be an easy task, even with the availability and development of non-pharmacological treatment options, including surgery, electrostimulation, diets and gene therapy, stem cell therapy, as well as palliative therapy, genetic diagnostics and neuroimaging techniques combined with machine learning technologies. Despite all the difficulties, the old concept of “rational polytherapy”, which appeared in the 19th century, combining drugs with different mechanisms of action and targets, can still improve the quality of life of patients [89]. Based on the available data and hypotheses, with all their evidence and challenges, more work is needed to support each of them and integrate current theories with the ultimate goal of developing more effective comprehensive treatments for epilepsy. The transport hypothesis and the target hypothesis are not mutually exclusive and both may be based on corresponding changes in the genome and epigenome. In the future, it is necessary to focus on the individual therapy of patients with refractory epilepsy, taking into account specific factors, such as the etiology of the disease, medical history, response to drugs, temporary patterns of refractoriness, as well as the multifactorial nature of drug resistance.

## Figures and Tables

**Table 1:** Some mediators involved in neuroinflammation and epilepsy.

Complement System	
C3, C4, Properdin, FH, C1Inh and Clu	Known as markers of epilepsy [32,33]. Components including C9, C8, C4-B are activated in patients. Consequently, the complement cascade is involved in the chronic epileptic phase in animals and humans. Complement activation can promote a sustained inflammatory response and destabilize neural networks involved in the pathological process [32]. In epilepsy, both classical (C1inh, C4) and alternative (FH, properdin, C3) pathways are damaged. These proteins allow to distinguish patients with well-controlled epilepsy from uncontrolled ones [31,32].
C3	Genetic polymorphisms in the promoter region obtained in patients suggest C3 role in the genetic predisposition to febrile seizures and epilepsy [31,32]. C3 deficient mice were found to be more resistant to seizures [32]. Serum C3 level is elevated in untreated patients compared to control and treated patients [32].
C1q and iC3b	The elevated levels of these proteins are registered in brain tissue samples from patients with drug-resistant epilepsy. C1q has been implicated in the pathological elimination of synapses in the context of schizophrenia and dementia. Elevated C1q and iC3b levels have been reported in human brain samples with focal cortical dysplasia. Thus, it can be assumed that aberrant complement activation occurs in patients with drug-resistant seizures [31,32].
Membrane Attack Complex (MAC)	MAC is recorded in activated microglia and neurons in the brain tissue of patients and animals with epilepsy. Sequential intrahippocampal injection of individual MAC proteins induces convulsions and neurodegeneration in rats [32].
Cytokines	
IL-1β	Elevated level of IL-1β suggests that inflammation is involved in the pathophysiology of epilepsy. In the CNS, IL-1β is mainly produced by activated microglia but also by neurons, astrocytes and oligodendrocytes. In a healthy brain, IL-1β is present at a low level, participating in the processes of sleep, learning, memorization and neuromodulation. In chronic and acute inflammatory processes in CNS, it plays both a useful and harmful role. IL-1β levels in the peripheral blood of patients may reflect the severity of seizures. It can inhibit gamma-aminobutyric acid (GABA)-mediated neurotransmission, inhibit glutamate uptake by astrocytes and modulate neuronal arousal. Inhibition by an IL-1RI antagonist or prevention of synthesis has a neuroprotective effect [41].
IL-2, IL-8, IL-18	In patients and animal models of epilepsy, increased expression levels of these cytokines in the brain are observed. They increase the excitability of neurons and thus are considered to be involved in epileptogenesis [25,41].
Arg1, IL-4 and IL-10	There is an increase of anti-inflammatory cytokines expression (Arg1, IL-4 and IL-10) by microglia in epilepsy [25]. IL-10 is usually characterized as an anti-inflammatory cytokine. In combination with transforming growth factor beta (TGF-ß), it inhibits a number of pro-inflammatory mediators, such as IL-1α, IL-1β, IL-6, IL-8, IL-12, IL-18, TNF-α and granulocytes, thus modulating glial activation. The anticonvulsant effect of IL-10 has been confirmed by studies in animal models [41].
IL-6	IL-6 is expressed by a number of brain cells, including astrocytes, microglia and neurons. IL-6 plays a controversial role in neuroinflammation, it can act as a pro-inflammatory cytokine, increasing the chemokine secretion and adhesion molecules, or inhibit TNF-α, reduce neurotoxicity, promoting differentiation and survival of neurons. IL-6 overexpression in CNS leads to aberrant hippocampal arousal, spontaneous seizures and neurodegeneration [25,41].
TNF-α	TNF-α probably plays a dual role as a pro- and anti-inflammatory cytokine, depending on the time, size, cell targets and signaling cascades involved, being both pro- and antiseizure [41].
TGF-β	Signaling of TGF-β has been shown to trigger seizures, neuronal hyperexcitability and epileptogenesis. Transcriptome analysis also confirms the role of TGF-ß signaling in epileptogenesis. Astrocytic transmission of TGF-ß signals induces excitatory synaptogenesis, which precedes the development of seizures [25].
NLRP3	The expression of the main component of inflammasomes (NLRP3) increases in the cerebral cortex of patients with refractory epilepsy. NLRP3 activates caspase-1, which leads to the processing of proinflammatory cytokines IL-1β and IL-18 [42].
Chemokines	
Fractalkin (FKN, CX3CL1)	This transmembrane chemokine is expressed by neurons of CNS. Several studies have shown its role in the epilepsy pathogenesis and concomitant cell death. Blocking of CX3CL1/CX3CR1 signaling pathway by antibodies reduces microglial activation and neurodegeneration caused by an electrical epileptic seizure in rodents [43].
CCL2	CCL2 expression is increased in the epileptic brain of humans and animals. Suppression of this chemokine can inhibit brain damage caused by seizures [41,44].
CCR7, CCR8, CCR9, CCR10	Production of these chemokines is suppressed in the hippocampus in animal models of epilepsy, the consequences of this suppression are not established yet [41].
CCL5, CCL19, CCL22, CXCL8	Elevated levels of these chemokines are observed in patients with epilepsy, traumatic brain injuries and in animal models of epilepsy [41].
Chemokine Receptor 7 (CXCR7)	CXCR7 is involved in the epilepsy pathogenesis and mediates the immune response in the brain. CXCR7 inhibition in the hippocampus had an antiepileptic effect on mice [45].

**Table 2:** Hypotheses of the Causes of Drug Resistance of Epilepsy.

Hypothesis	Description	Supportive	Non-Supportive
Pharmacokinetic Hypothesis	Overexpression of drug efflux vectors in peripheral organs lowers the levels of anticonvulsants.	Transporter overexpression in the BBB and neurons does not explain the decrease in drug concentration in clinical observation.	The animal studies do not support the pharmacokinetic hypothesis.
Transport Hypothesis	Overexpression of drug efflux vectors in the BBB lowers the levels of anticonvulsants.	Multidrug resistance proteins are overexpressed in the endothelial cells of the brain capillaries and in the astrocytes in drug-resistant epilepsy	This theory is only supported by animal studies, not in human tissues.
Neural Network Hypothesis	Neuron degeneration and the synaptic network remodeling leads to the brain’s seizure control system suppression and drug access to targets restriction.	Cortical dysplasia is often associated with drug-resistant epilepsy. The pathological neural networks underlie focal drug-resistant epilepsy.	Some patients with cortical dysplasia and changed neural networks do not have drug resistance. After temporal lobe resection part of patients do not respond to ASMs.
Intrinsic Severity Hypothesis	The neurobiological factors affect both the severity of epilepsy and drug resistance.	This theory is supported by clinical reports and transcriptome analysis of human hippocampal tissues.	It is shown that drug resistance does not depend on the number and severity of seizures before treatment.
Genetic Variants Hypothesis	Genetic polymorphisms are associated with pharmacodynamics, metabolic pathways, enzymes, ion channels and neurotransmitter receptors, block drug binding, metabolism and transport lead to drug resistance development.	It was found that polymorphisms were associated with various types of epilepsy and genetic changes occurred in patients with untreatable seizures.	
Epigenetic Hypothesis	The epigenome changes can play a role in drug resistance patterns.	Manipulations with microRNAs can influence seizures and the course of epilepsy in experiments using laboratory animals.	It is difficult to separate cause from effect and significance from epiphenomena, especially on humans.
Target Hypothesis	Quantitative and qualitative changes in potential-dependent ion channels and neurotransmitter receptors lead to a decrease in drug sensitivity and drug resistance development.	Dravet syndrome studies, studies of the effectiveness of carbamazepine and phenytoin support this hypothesis.	Patients with drug-resistant epilepsy usually do not respond to drugs of different classes with different mechanisms of action.
Hypothesis of Neuroinflammation	Neuroinflammation can induce BBB dysfunction and up-regulate Pgp expression in drug-resistant epilepsy.	In the experiment and in clinic, it was found that the permeability of the BBB increases in foci of chronic epilepsy. Many studies show increase of cytokines in brain and in plasma in patients with drug-resistant epilepsy and in animal models.	
